# Supplementary material for: Exploring the knowledge and attitudes of Cameroonian medical students towards global surgery: A web-based survey
Source: PLoS One. 2020 Apr 30;15(4):e0232320. doi: 10.1371/journal.pone.0232320 (PMC7192476; doi:10.1371/journal.pone.0232320)
Supplement: S1 File — (PDF) [file pone.0232320.s002.pdf]

Knowledge and Attitudes of Medical Students Towards Global Surgery in Cameroon/Connaissances et attitudes des étudiants en médecine à l'égard de la chirurgie globale au Cameroun

Nous vous invitons à participer à une enquête en ligne sur les connaissances et les attitudes des étudiants en médecine et des professionels de santé à l'égard de la chirurgie globale au Cameroun. Il s'agit d'un projet de recherche mené par le Groupe de travail national du réseau estudiantin international de chirurgie (InciSioN) - Cameroun. Ceci devrait vous prendre environ 15 minutes.

Votre participation à cette enquête est volontaire et vous pouvez refuser de participer ou décider de quitter l'enquête à tout moment sans pénalité. Vous êtes libre de refuser de répondre à toutes les questions auxquelles vous ne souhaitez pas répondre.

Vous ne recevrez aucun avantage direct de votre participation à cette étude. Cependant, vos réponses nous aiderons à en apprendre plus sur les connaissances, les attitudes et les pratiques des étudiants en médecine et des professionnels de santé à l'égard de la chirurgie globale.

Il n'y a pas de risques prévisibles associés à votre participation à cette étude autres que ceux rencontrés dans la vie quotidienne.

Vos réponses à cette enquête seront collectées sur Google Forms, stockées électroniquement et protégées par un mot de passe. Google Forms ne collecte pas d'informations d'identification telles que votre nom, votre adresse e-mail ou votre adresse IP donc vos réponses resteront anonymes. Personne ne pourra vous identifier ni identifier vos réponses, et personne ne saura si vous avez participé ou non à cette enquete.

Si vous avez des questions concernant ce projet, veuillez contacter InciSioN Cameroon.

En poursuivant, vous acceptez de participer à cette étude et confirmez que

• Vous avez lu les informations ci-dessus
• Vous acceptez de participer volontairement
• Vous avez 18 ans ou plus

You are invited to participate in a web-based online survey on the Knowledge and Attitudes of Medical Students and Healthcare Workers Towards Global Surgery in Cameroon. This is a research project conducted by the International Student Surgical Network (InciSioN) National

Working Group - Cameroon. It should take approximately 15 minutes to complete.

Your participation in this survey is voluntary. You may refuse to take part in the research or exit the survey at any time without penalty. You are free to decline to answer any particular question you do not wish to answer for any reason.

You will receive no direct benefits from participating in this research study. However, your responses may help us learn more about the knowledges, attitudes and practices of medical students and health professionals towards global surgery.

There are no foreseeable risks involved in participating in this study other than those encountered in day-to-day life.

Your survey answers will be collected on Google Forms and stored in a password protected electronic format. Google Forms does not collect identifying information such as your name, email address, or IP address. Therefore, your responses will remain anonymous. No one will be able to identify you or your answers, and no one will know whether or not you participated in the study.

If you have questions at any time about the study or the procedures, you may contact InciSioN Cameroon

By proceeding with this questionnaire you agree to participate in this study and confirm that

- You have read the above information
- You voluntarily agree to participate
- You are 18 years of age or older

*Required

1.

Mark only one oval.

- D'accord/Agree
- Non/Disagree

Données sociodémographiques et définition de chirurgie globale/Sociodemographic characteristics and Global Surgery definition

2. Sexe/Sex

Mark only one oval.

- Homme/Male
- Femme/Female
- Je ne souhaite pas divulguer/I don't want to disclose

3. Age

____

4. Université/University

Mark only one oval.

- FMSB
- ISTM
- UdM - ISSS
- FMSP
- FHS - Bamenda
- FHS - Buea
- Other: ________________________

5. Lieu de résidence/Residency address

Mark only one oval.

- Extreme Nord/Far North
- Nord/North
- Adamaoua/Adamawa
- Ouest/West
- Nord Ouest/North West
- Est/East
- Littoral
- Sud Ouest/South West
- Centre
- Sud/South

6. Avez-vous déjà entendu parler de ou lu sur la chirurgie globale?/Have you previously heard or read about global surgery?

Mark only one oval.

- Oui/Yes
- Non/No

7. Si oui, en avez-vous entendu parler?/If yes, where did you hear about it?

Tick all that apply.

- [ ] Twitter
- [ ] WhatsApp
- [ ] Facebook
- [ ] Télévision/Television
- [ ] Une connaissance/An acquaintance
- [ ] A l'hopital/At the hospital
- [ ] A l'Université/At School
- [ ] Autre/Other

8. Quelle est selon vous la définition de la chirurgie globale?/How would you define global surgery?

____

Expériences de chirurgie globale/Global surgery experience

La chirurgie globale utilise la recherche, l'éducation et le plaidoirie pour offrir un accès universel à des soins chirurgicaux, obstétricaux et anesthésiques sûrs et abordables en temps opportun./Global surgery uses research, education and advocacy to bring universal access to safe, timely and affordable surgical, obstetric and anesthesia care.

9. Avez-vous déjà participé à un événement de chirurgie globale?/Have you ever attended a global surgery event?

Mark only one oval.

- ◯ Oui/Yes
- ◯ Non/No

10. Si oui, où avez-vous participé à cet événement?/If yes, where did you participate in this event?

Tick all that apply.

- ☐ En ligne/Online
- ☐ Au Cameroun/In Cameroon
- ☐ A l'étranger (en Afrique)/Abroad (in Africa)
- ☐ A l'étranger (en dehors de l'Afrique)/Abroad (out of Africa)

11. Avez-vous déjà participer à une étude de chirurgie, d'anesthésie ou d'obstétrique?/Have you ever participated in a surgery, anesthesia or obstetrics study?

Mark only one oval.

- ◯ Oui/Yes
- ◯ Non/No

12. Si oui, quel était votre role/If yes, what was your role?

Tick all that apply.

- [ ] Investigateur/Investigator
- [ ] Collaborateur/Collaborator
- [ ] Répondant/Respondent
- [ ] Collecteur de données/Data collector
- [ ] Other: ______________________

Connaissances en matière de chirurgie gloable/Global Surgery knowledge

13. Lequel des organismes/concepts qui suivent connaissez-vous?/Which of the following have you heard or do you know about?

Tick all that apply.

- [ ] Lancet Commission on Global Surgery (LCoGS)
- [ ] Disease Control Priorities (DCP 3)
- [ ] InciSioN
- [ ] Global Surgery Student Alliance (GSSA)
- [ ] Global Anaesthesia, Surgery and Obstetric Collaboration (GASOC)
- [ ] G4 Alliance
- [ ] Mercy Ships
- [ ] Gender Equity in Global Surgery
- [ ] Program in Global Surgery and Social Change (PGSSC)
- [ ] College of Surgeons of East, Central and Southern Africa (COSECSA)
- [ ] Operation Smile
- [ ] Kids OR
- [ ] Lifebox
- [ ] Safe surgery 2020
- [ ] National Surgical, Obstetrics and Anesthesia Plan (NSOAP)
- [ ] Consortium of Universities in Global Health (CUGH)
- [ ] Bellwether procedures
- [ ] Indicateurs de chirurgie globale du Lancet/Lancet global surgery indicators
- [ ] Other: ______________________

14. Lequel des indicateurs suivants de la Commission Lancet sur la chirurgie globale connaissez-vous?/Which of the following Lancet Commission on Global Surgery indicators are you familiar with? *

Tick all that apply.

- [ ] Accès à la chirurgie en temps opportun/Access to timely surgery
- [ ] Main d'œuvre chirurgicale spécialisée/Specialist surgical workforce
- [ ] Volume opératoire/Surgical volume
- [ ] Taux de mortalité périopératoire/Perioperative mortality rate
- [ ] Risque de dépenses appauvrissantes/Risk of impoverishing expenditure
- [ ] Risque de dépenses de santé catastrophiques/Risk of catastrophic expenditure

15. Les assertions qui suivent sont-elles vraies ou fausses?/State whether the following assertions are true or false

Mark only one oval per row.

	Vrai/True	Faux/False	Je ne sais pas/I don't know
Les maladies chirurgicales tuent plus de personnes que les maladies non transmissibles/Surgical diseases kill more people than noncommunicable diseases	⬭	⬭	⬭
Les traitements chirurgicaux coûtent beaucoup plus que les traitement médicaux/Surgical treatments cost a lot more than medical treatments	⬭	⬭	⬭
Les pathologies chirurgicales ne constituent pas un problème majeur de santé publique au Cameroun/Surgical pathologies are not a major public health problem in Cameroon	⬭	⬭	⬭
Les pathologies chirurgicales entraînent des handicaps et contribuent à l'appauvrissement de notre nation/Surgical pathologies lead to handicaps and contribute to the impoverishment of our nation	⬭	⬭	⬭
Le manque criard de spécialiste impose la formation de non-spécialistes et la délégation de taches et des responsabilités à ces personnes/The lack of specialist imposes the training of non-specialists and the delegation of tasks and responsibilities to these people	⬭	⬭	⬭
Le manque de couverture santé universelle contribue à l'appauvrissement des Camerounais/Lack of universal health coverage contributes to impoverish Cameroonians	⬭	⬭	⬭
Toutes les 2 secondes, une personne	⬭	⬭	⬭

décède des suites d'une pathologie chirurgicale dans le monde/Every 2 seconds, a person dies from a surgical pathology in the world			
9 des 13 objectifs du développement durable sont affectés par les pathologies chirurgicales/9 of the 13 sustainable development goals are affected by surgical pathologies	◯	◯	◯
Les femmes césarisées en Afrique ont plus de chance de mourir que celles qui ne sont pas en Afrique/Ceasarized women are more likely to die if they are from Africa	◯	◯	◯
5 milliards de personnes n'ont pas accès à des soins chirurgicaux de qualité et à temps/5 billion people do not have access to quality surgical care on time	◯	◯	◯

Attitudes à l'égard de la chirurgie globale/Attitudes towards global surgery

16. Les éléments suivants doivent être développé afin de mieux prendre en charge un plus grand nombre de Camerounais en chirurgie, obstétrique et anesthésie/The following should be developed in order to increase surgical, obstetric and anesthetic access and reach more Cameroonians

Mark only one oval per row.

	Tout à fait d'accord/Strongly agree	D'accord/Agree	Indifférent/Neutral	Pas d'accord/Disagree
Plus de financement/More funding	⬭	⬭	⬭	⬭
Plus de personnel/More personnel	⬭	⬭	⬭	⬭
Lutte contre la corruption/Fight against corruption	⬭	⬭	⬭	⬭
Bonne gouvernance/Good governance	⬭	⬭	⬭	⬭
Plus de partenariats public-privé/More public-private partnerships	⬭	⬭	⬭	⬭
Plus de partenariats avec les pays occidentaux/More partnerships with Western countries	⬭	⬭	⬭	⬭
Plus de partenariats avec les pays Africains/More partnerships with African countries	⬭	⬭	⬭	⬭
Plus de structures de santé	⬭	⬭	⬭	⬭

primaire/More primary health structures				
Plus de structures sanitaires spécialisées/More specialized health structures	◯	◯	◯	◯
Plus grande participation des populations locales dans les décisions sanitaires/Greater involvement of local populations in health decisions	◯	◯	◯	◯

This content is neither created nor endorsed by Google.

Google Forms
