# Supplementary material for: Exploring the knowledge and attitudes of Cameroonian medical students towards global surgery: A web-based survey
Source: PLoS One. 2020 Apr 30;15(4):e0232320. doi: 10.1371/journal.pone.0232320 (PMC7192476; doi:10.1371/journal.pone.0232320)
Supplement: Attachment — Submitted filename: Response to Reviewers.docx [file pone.0232320.s003.docx]

**REBUTTAL LETTER**

Dear Editor and Dear reviewers,

We wish to thank you for taking the time to review our manuscript. The suggestions you made and the questions you raised were very helpful and accurate. We have addressed your comments (in red in this document) and we feel this has substantially improved the quality of our manuscript. We look forward to reading your comments.

**Additional Editor Comments:**

There are significant issues identified by the reviewers. I want to highlight two

1. This study would not be appropriate for publication in PLOS ONE unless the application of the findings would be wider than Cameroon.

Re: We agree with the Editor that the previous discussion had a narrow scope. We have rewritten the discussion so it applies to a broader scope. We discuss the similarities between our findings and US studies and we go on to discuss the implications for low- and middle-income countries.

2. If the authors would want to have the study published in this journal, I would suggest redoing the questionnaire, and showing clearly how questions were asked. They could then administer it to a homogenous group such as all final year medical students in the country, and find a way to achieve a high response rate (preferably 80% or above).

Re: Dear Editor, we thank you for your remarks. We have re-administered the survey to final year medical students. To achieve a greater response rate, we worked with class delegates and sent weekly reminder messages in the WhatsApp group. As a result, we have rewritten the methods and results sections.

Journal requirements:

When submitting your revision, we need you to address these additional requirements.

1. Please ensure that your manuscript meets PLOS ONE's style requirements, including those for file naming. The PLOS ONE style templates can be found at http://www.journals.plos.org/plosone/s/file?id=wjVg/PLOSOne_formatting_sample_main_body.pdf and http://www.journals.plos.org/plosone/s/file?id=ba62/PLOSOne_formatting_sample_title_authors_affiliations.pdf

Re: We read and applied the style requirements to the body and to the author affiliations (see title page lines 1-31).

2. Please include additional information regarding the survey or questionnaire used in the study and ensure that you have provided sufficient details that others could replicate the analyses.

For instance, if you developed a questionnaire as part of this study and it is not under a copyright more restrictive than CC-BY, please include a copy, in both the original language and English, as Supporting Information.

Re: Thank you for pointing this out. The survey form has been submitted along with this rebuttal and we have included additional information about the survey in the methods section (lines 109-121).

3. Please state whether you validated the questionnaire prior to testing on study participants.

Please provide details regarding the validation group within the methods section.

Re: The questionnaire was assessed by three global surgery experts. They suggested modifications and following edits they established face validity. We ran a pilot test with 10% (20) of the total target population. This has been added to the methods section (Lines 109-113)

4. In your Methods section, please include additional information about your dataset and ensure that you have included a statement specifying whether the collection method complied with the terms and conditions for the website.

Re: The data sources have been acknowledged in the methods (line 113) and a statement specifying that data collection complied by the terms and conditions was added (lines 129-130)

**Reviewers' comments:**

**Reviewer's Responses to Questions**

**Comments to the Author**

**5. Review Comments to the Author**

Please use the space provided to explain your answers to the questions above. You may also include additional comments for the author, including concerns about dual publication, research ethics, or publication ethics. (Please upload your review as an attachment if it exceeds 20,000 characters)

Reviewer #1: The paper reads well but is primarily a discussion on global surgery. There is clearly a need to develop surgical services in Cameroon, but does the workforce need to advocate global surgery or to focus on developing surgical services in Cameroon itself? The latter is likely more important. There should be details included of the plans for Cameroon to develop their surgical services and whether the government is guided by global health targets.

Re: Dear reviewer, thank you for this valuable comment. The public health system in Cameroon is very centralized: key decisions are made at the Ministry of Health (MoH) level based on information gathered at the peripheral level (district hospitals). The information gathered peripherally is relayed to the central MoH by multiple mid-level administrative bodies. As a result, bureaucracy delays responses from the MoH and leads to a fragmentation of the public health system.

Another problem is the earmarking of funds for diseases such as HIV/AIDS, Malaria and Tuberculosis. This has created a disparity between these high-profile diseases and other diseases. For example, most performance-based financing indicators are focused on infectious diseases, non-surgical maternal health and non-surgical child health. Consequently, the provision of care is skewed towards the incentivized diseases to the detriment of the other diseases. This gives the impression that the burden of the non-incentivized diseases is small or negligible. We believe the workforce can advocate for surgically amenable diseases if they advocate for the non-incentivized diseases. They have a role to play in surgical services delivery in Cameroon but at the moment their capacity is limited by non-recognition of the burden of surgically amenable diseases.

The contribution of Mercy Ships is well made through the lead in training and providing a vital service for the local community.

I believe this paper is worthy of publication but more as a discussion of the need for countries (like Cameroon) to work towards achieving Global Surgery targets. The questionnaire is more of a side discussion.

Statistics are included in the paper but do not add to the analysis and should be removed.

Re: Dear reviewer, we share your opinion on the quality of the survey. Following advice from the academic editor, we re-administered the survey to a more homogenous population (final year medical students). We ran summary descriptive analysis and non-parametric bivariate analysis. As a result, we have rewritten the results and discussion sections to reflect this change.

Reviewer #2: I am interested that the lead researcher appears to be based in DR Congo and ethics was approved there, although the study was conducted in Cameroon. This seems to conflict with line 117 where the manuscript states that local ethical approval was obtained.

Re: Dear reviewer, thanks for taking the time to review our article. We found your comments accurate and beneficial. We had obtained IRBs at the academic institutions of the authors in Cameroon and DR Congo. We have attached the IRB obtained in Cameroon to this submission.

The introduction is nicely constructed and builds to the purpose of this study.

It would be very helpful to have some workforce data for Cameroon. Eg what percentage of doctors registered in Cameroon work in anaesthetic, obstetric or surgical disciplines? <http://cm-minsante-drh.com/site/images/stories/Rapport_general_du_recensement01_12_2011_misenforme_FINAL05122001.pdf>

Re: Thank you for your advice and kind words. We agree with you that having workforce data will make for a better read. We have included data on the surgical, obstetric and anesthesia workforce in the manuscript (lines 101-102).

Line 92 I am unsure what the authors mean when they say that global surgery in Cameroon is ‘led’ by InciSioN? Do they mean that InciSioN is one of the leading pressure groups promoting global surgery?

Re: Dear reviewer, we agree with you. We meant that InciSioN is one of the leading pressure groups promoting global surgery. We opted to exclude this statement as well as most mentions of InciSioN in the manuscript. As you rightfully noted our paper was monofocused.

Line 101-102: I feel that the authors could emphasize the wider potentials for the study. This study would not be appropriate for publication in PLOS ONE unless the application of the findings would be wider than Cameroon.

Re: Dear reviewer, we have rewritten our discussion to emphasize the wider potential of the study in the United States and low- and middle-income countries.

In Methods the authors first describe how they designed their survey line107. This section is brief and the reader only really knows which literature was read.

Re: Dear reviewer, thanks for your remark. Per your recommendation we have rewritten the methods and given additional details.

The questionnaire itself needs to be seen so that readers can see how questions were expressed, and could repeat the study in their own country or at a different time.

Re: We apologize for this mistake. We have added the questionnaire to the resubmission.

The authors then describe how they found their participants for the study. Their description is not very clear – either in terms of how they selected or accessed their target audience. They say that they used a convenience non-probability sampling method but do not reference this or explain how it worked, line 112.

They do not state how many people received the questionnaire, and hence the response rate is not evident.

It is very hard to know how representative their sample is likely to be.

Re: Dear reviewer, we have addressed recruitment, response rate and representativeness in our methods (lines 115-121).

In line 112,113 I am unsure why they chose medical Students and nurses and doctors including surgeons. It seemed to be anyone who was willing to complete the survey. The study would have more clarity if it was a single target audience. It is hard to imagine the respondents did not self-select as those interested in the topic. The groups sampled cross professions and training/trained demographics. For the medical students we are not told in which year of study they are currently. Clearly a first year medical student would have less chance of understanding global surgery than a final year student. We are not told if there is teaching on this subject in the medical student curriculum, and if so in which year of study it is taught?

The forms were anonymous but the authors say that they omitted members of the InciSioN group – how did they do this?

Re: Following your remarks and those of the academic editor we re-administered the survey to a homogenous population of seventh-year medical students. Our previous response rate had been affected by the conflicts in the North West and South West regions of Cameroon – where two of the six medical schools are located. We have been able to reach a much wider audience, ensuring generalizability.

Results

Line 121 there were only 45 respondents. We are not informed how many medical students are present in Cameroon, or indeed health care workers.

Re: Dear reviewer, we have re-administered the survey and rewritten both the methods and results to address this issue.

Line 129: do the authors mean most popular (well thought of) or popular (well known).

In the table:

Health professionals presumably means 18 doctors and one nurse?

Re: Dear reviewer, we have re-administered the survey to final year medical students and rewritten both the results and discussion.

The question ‘do you know about ‘global surgery’ appears confusing, and might be better expressed ‘have you previously heard of the term ‘global surgery’?’

Re: We agree with you esteemed Dr. The question is phrased as you suggested in our survey form which has been submitted along with this response.

For all the summarised section results it is unclear whether or not these are based on open or closed questions. For example were the organisations named and the respondents stated whether or not they had heard of them, or were they free text responses conceived by respondents?

Re: We have submitted the survey form along with this letter. The organizations were listed and the respondents could list an organization if it was not on the list.

Line 144 some results are given in the text but not in the table whilst others are in the table and repeated in the text. This needs to be consistent.

Re: Thanks Dr. We have attended to this in our new Table 1 (line 145).

Having not seen the questionnaire I am unsure whether any questions explored the place of trauma and injury in surgically treatable disease. This is particularly relevant to the perception of surgically treatable disease as a public health issue.

Re: We agree with you. We opted to limit our questions to the basic concepts of global surgery because there are other key concepts such as emergency obstetric care and emergency laparotomies that are just as deserving but would have lengthened the survey.

Discussion

The discussion is quite long but rather mono-focussed.

There is no comment on similar literature.

Re: Thanks for your insightful comment. We have rewritten the discussion.

There is some discussion regarding why respondents responded as they did.

Much of the focus seems to bear on proposed solutions to spread the word regarding global surgery, and a bit of publicity for InciSioN Cameroon. It would be helpful if the authors explored the issue of current knowledge and understanding of global surgery without such particular emphasis on InciSioN.

Re: We have rewritten the discussion to be less monofocused.

Conclusion

Line 225 the authors state their laudable organisational aims. However, it would be helpful if the manuscript could be expressed a little more objectively and scientifically.

Re: You are right dear reviewer. We have rewritten the conclusion to be less passionate and more scientific.

Figure

The authors show the type and distribution of respondents. It would be helpful to know where the medical schools are distributed and how many medical students are present in each.

Re: Following your advice, we have redesigned the figure to show the location of the medical schools.

Reviewer opinion:

The authors have an original and interesting research concept. The authors are to be thanked for raising this debate and taking time to undertake this survey and prepare the manuscript.

I agree with them that this subject is of importance in the Universal health Coverage debate. They also clearly have great passion for their subject material. They have presented some interesting findings, and make some interesting proposals.

The big challenge here is the methodology. Details of this are noted in my comments. The likelihood of self-selection of respondents and the heterogeneity of the sample render the results impossible to analyse or to use for meaningful conclusions. The sample size is relatively small. Even with improved explanation of the methodology used, I think there would be too many concerns on methodology to enable the results to be published.

If the authors would want to have the study published in this journal, I would suggest redoing the questionnaire, and showing clearly how questions were asked. They could then administer it to a homogenous group such as all final year medical students in the country, and find a way to achieve a high response rate (preferably 80% or above).
