# Supplementary material for: Exploring the knowledge and attitudes of Cameroonian medical students towards global surgery: A web-based survey
Source: PLoS One. 2020 Apr 30;15(4):e0232320. doi: 10.1371/journal.pone.0232320 (PMC7192476; doi:10.1371/journal.pone.0232320)
Supplement: Attachment — Submitted filename: Response to Reviewers.docx [file pone.0232320.s004.docx]

Response to reviewers

Reviewer #1: The authors should be congratulated for a thorough reworking of the paper and their commitment demonstrated by carrying a wider survey. I believe this paper is a useful contribution to the literature and I hope the aims of there authors to raise the profile of surgical care in Cameroon will be met.

R:

Dr Gardner, this was possible thanks to you. Your comments greatly improved our manuscript.

2 minor comments:

Line 39: only medical students completed the questionnaire

R: We have corrected the sentence. (Line 39-40)

Line 148: typo- remove 'were'

R: Thanks. The typo has been corrected. (Line 151)

Reviewer #2: Exploring the knowledge and attitudes of Cameroonian medical students towards global surgery: a web based survey

Reviewer comments:

Answering questions raised: the authors have been at pains to answer the questions and respond to the issues raised in the review of the first manuscript. They have gone to the lengths of completely re-administering the questionnaire and rewriting the whole manuscript. Effectively they have gone to the length of starting from scratch with the same concept but delivered with a greatly enhanced methodology.

R:

Thank you for your comments and kind words. You made this process enjoyable and rewarding. We learnt a lot.

Abstract: in results I would say 58% of respondents were male – rather than ‘most’.

R:

Thank you for pointing this out. We have made the changes suggested. (Lines 50-51)

The authors say that the mean knowledge level was 47%. The terminology ‘knowledge level’ is difficult for the reader to understand or quantify. The terminology assuming ‘numbers of roles’ is not easily understood by the reader.

R:

We have reworded the text and we hope it is clearer for the audience. (Line 53-57)

Introduction:

This is nicely constructed with quotations which highlight the importance of the topic. It builds towards the purpose of the study. There is a slightly abrupt transition from the global surgical need to the knowledge and opinions of medical students. Perhaps a bridging sentence such as ‘since Medical Students form the national potential in workforce capacity development, their attitudes towards global surgery are of paramount importance.’

R:

Thank you. We have made changes to the introduction. (Lines 105-107)

Methodology:

This description is now succinct and much easier to understand than previously. The target group is homogenious and quantifiable.

A pilot study was used to hone the quality of the questionnaire.

I would still really like to see the survey rather than just a link to it. This would be one of my main recommendations.

R:

We are sorry for the inconvenience caused. Following your initial comments, we had submitted the questionnaire as a supplemental file. However, it seems you did not get the file. We have resubmitted the questionnaire.

Results:

Since the aim is to look at Medical student knowledge and attitude, I am unsure as to why the results are divided by sex? The aim was not to look as sex differences in attitude and knowledge. No gross differences emerge, so why not simply present the results for all respondents and say that no significant differences were observed based on the sex of the student?

R:

Thank you for your comments. Although the sex disparities were not statistically significant, the differences between the two genders were important. It is possible that we were not powered to detect the differences in gender. Surgery and global surgery are fields dominated by men and we felt it was critical to report on the gender differences (Odds ratio of female/male 1.61 and 1.40). We presented these results to highlight this too. With your permission we wish to keep these results.

I am not sure that figure 1 adds a huge amount. The main question seems to be - of the six medical schools in the country, what was the variation in participation rate? This may affect the generalizability of the findings.

R:

Dear reviewer, we added figure 1 at the request of reviewer 1 who felt it was important to show the distribution of participants.

The results summarized in the text lines 148-174 are useful and interesting.

R:

Thank you

Line 167 assuming numbers of roles is not understood to this reviewer and therefore probably not to most readers. Can this be defined?

R:

Dear reviewer, thank you for your comments. We have reworded the sentence. (Lines 170-172)

Figure 2 is quite useful in allowing the reader to compare the strength of opinion relating to the responses with Likert grades.

R:

Thank you.

Discussion:

The discussion is much improved from last time with some comparison to other studies and broader assessment of the impact of educating medical students regarding global surgery.

R:

We are honored by your comments and grateful for your help.

I am interested that the authors do not recommend a change in the undergraduate curriculum to include at least on lecture on global surgery, and perhaps another on universal health access? What they seem to have discovered is that social media and external events are moderately effective in filling a gap in the curriculum. It seems reasonable that they should advocate for that gap to be filled.

R:

Thank you for pointing this out. We have added your suggestion to the discussion. (Line 187-188)

When discussing the limitations the authors focus on survey uptake and whether the sample is representative. In fact this has become a strength of the study with more than 80% response rate.

Other limitations would include:

Variations in understanding the questions; limitations of areas of questioning encompassed by the survey; unproven generalizability of these results to other lmics.

R:

We have added these to our limitations. (Lines 227-229)

Conclusion:

The conclusion is a little wordy. The last sentence is a good one but perhaps should say ‘in global surgical education and activities’.

R:

We have rewritten the conclusion per your recommendation. (Conclusion)

Overall reviewer opinion:

This is a vastly improved and totally reworked manuscript. It is an important area of research. It is now a coherent study. I would recommend it as being suitable for publication subject to a few minor edits.

R:

This would not have been possible without your help. Thank you for your patience and assistance all throughout this process.
